# Defining a Radiomic Response Phenotype: A Pilot Study using targeted therapy in NSCLC

**DOI:** 10.1038/srep33860

**Published:** 2016-09-20

**Authors:** Hugo J. W. L. Aerts, Patrick Grossmann, Yongqiang Tan, Geoffrey G. Oxnard, Naiyer Rizvi, Lawrence H. Schwartz, Binsheng Zhao

**Affiliations:** 1 Departments of Radiation Oncology, Brigham and Women’s Hospital, Harvard Medical School, Boston, MA, USA; 2 Departments of Radiology, Brigham and Women’s Hospital, Harvard Medical School, Boston, MA, USA; 3 Departments of Biostatistics & Computational Biology Dana-Farber Cancer Institute, Brigham and Women’s Hospital, Harvard Medical School, Boston, MA, USA; 4 Departments of Radiology Columbia University College of Physicians and Surgeons and New York Presbyterian Hospital, New York, NY, USA; 5 Department of Medicine, Departments of Lowe Center for Thoracic Oncology, Dana-Farber Cancer Institute, Boston, MA, USA; 6 Department of Medicine, Division of Oncology Columbia University College of Physicians and Surgeons and New York Presbyterian Hospital, New York, NY, USA

## Abstract

Medical imaging plays a fundamental role in oncology and drug development, by providing a non-invasive method to visualize tumor phenotype. Radiomics can quantify this phenotype comprehensively by applying image-characterization algorithms, and may provide important information beyond tumor size or burden. In this study, we investigated if radiomics can identify a gefitinib response-phenotype, studying high-resolution computed-tomography (CT) imaging of forty-seven patients with early-stage non-small cell lung cancer before and after three weeks of therapy. On the baseline-scan, radiomic-feature Laws-Energy was significantly predictive for EGFR-mutation status (AUC = 0.67, *p* = 0.03), while volume (AUC = 0.59, *p* = 0.27) and diameter (AUC = 0.56, *p* = 0.46) were not. Although no features were predictive on the post-treatment scan (*p* > 0.08), the change in features between the two scans was strongly predictive (significant feature AUC-range = 0.74–0.91). A technical validation revealed that the associated features were also highly stable for test-retest (mean ± std: ICC = 0.96 ± 0.06). This pilot study shows that radiomic data before treatment is able to predict mutation status and associated gefitinib response non-invasively, demonstrating the potential of radiomics-based phenotyping to improve the stratification and response assessment between tyrosine kinase inhibitors (TKIs) sensitive and resistant patient populations.

The response of tumors as measured on imaging is historically one of the oldest biomarkers used in drug discovery and clinical practice. Response has been assessed by measuring tumor burden with a number of surrogates such as unidimensional measurements according to the RECIST criteria^1^ bidimensional measurements as defined by the World Health Organization^2^. The purpose of these criteria as well as the modifications to the criteria are twofold; first, to enhance the ability of the biomarker to be prognostic or predictive and/or to improve the accuracy and reproducibility of the biomarker. Tumor burden and change in tumor burden during therapy, as measured at imaging, has been demonstrated to have value as a biomarker^3^.

However, medical imaging can provide more information about the tumor phenotype, beyond volumetric measurements; a process referred to as image-based phenotyping. Medical imaging is intuitively very suitable as a biomarker source to predict treatment response, as it is able to visualize and quantify time series of disease processes in a non-invasive way in individual patients. The characterization of quantitative imaging features which reflect tumor biology, physiology and tumor phenotype is increasingly being explored. Radiomics is the study of these quantitative features and their correlation with tumor phenotypes^4^,^5^,^6^. For example, recent studies have used CT-based radiomic signatures to successfully predict overall survival, disease free survival, and distant metastases in lung cancer^4^,^7^,^8^. Other examples have demonstrated that an imaging feature which could be quantified, for example, the percentage of ground-glass opacity (GGO) volume, is significantly higher in patients with the exon 21 missense mutation than in tumors with other EGFR mutation status^9^. This is thought be related to the fact that exon 21 missense mutation was significantly more frequent in lepidic predominant adenocarcinomas^10^. While these quantitative imaging features are under investigation in many cancers for their correlation with tumor phenotype and mutational status there is preliminary evidence to suggest that there may be an association between these features and both clinical outcomes and the underlying genomic signatures in lung cancer^4^,^8^,^11^.

In this pilot study, we look for the first time at the value of quantitative radiomic imaging features, in addition to tumor burden which was previously studied, for predicting known sensitizing EGFR mutations associated with Gefitinib response, to understand the relationship between imaging features and mutational status at baseline and especially with change in therapy in patients with and without the sensitizing mutation.

## Material and Methods

This is a re-analysis of an existing dataset; imaging and tissue data were obtained prospectively as an exploratory analysis within a phase II trial of neoadjuvant Gefitinib in patients with NSCLC^12^. Correlation results of early diameter and volume changes with EGFR mutation status was published previously^3^. As described previously^3^, all experiments were performed in accordance with relevant guidelines and regulations, and approved by the institutional review board (IRB) at Columbia University College and New York Presbyterian Hospital. Also, informed consent was obtained from all subjects included in this study.

### Clinical data

At time of resection, tumor tissue was snap frozen in liquid nitrogen and stored in a −80 °C freezer. Representative areas of these specimens were pathologically reviewed to confirm the diagnosis and presence of tumor. Genomic DNA was analyzed for the most common EGFR-sensitizing mutations (exons 19 and 21) using previously described PCR-based methods^13^,^14^. EGFR wild-type (WT) tumors were also tested for KRAS mutations, which were found in a non-overlapping subset of lung adenocarcinomas that have been found to be resistant to EGFR tyrosine kinase inhibitor therapy^15^. If no EGFR or KRAS mutations were found, then the remaining EGFR exons were assessed by standard dideoxynucleotide sequencing. Selected specimens that were found to be EGFR/KRAS WT were submitted for more detailed mutational testing using mass spectrometry.

### Tumor imaging and measurement

Baseline computed tomography of each patient was done within 2 weeks before gefitinib initiation. A follow-up computed tomography scan was done using the same imaging acquisition technique about three weeks post therapy, before surgery. Non–contrast enhanced diagnostic chest computed tomographies were done with a LightSpeed 16 scanner (GE Medical Systems) during a breath hold. High-resolution images with 1.25-mm slice thickness and lung kernel were reconstructed^3^. Three patients were excluded because 1.25-mm slice thickness reconstructions were not collected as required by protocol (two patients) and delineation of lesion contour did not reach radiologists’ consensus during the data review of this study (one patient), leaving 47 of 50 patients remained in our analysis. Tumor contours were semi-automatically delineated using a three-dimensional segmentation algorithm^3^,^16^.

### Quantitative Radiomics analysis

In this work, we extracted 183 radiomic features from both baseline and follow up scan images that were resampled down to 0.25 mm resolution in all three directions. The definitions of these features are provided in Supplemental 1. The Delta dataset was defined as the pre-treatment radiomic feature values minus the post-treatment values. On the Delta dataset, the 15 most variant features were selected using the coefficient of variation. From this set, highly correlated features were removed who had a mean correlation of higher than 0.95. This procedure yielded 11 independent features; we added Volume and maximum diameter for comparison, resulting in 13 features in total. A correlation matrix for these features was calculated using Spearman rank statistic. For every dataset, the area under the curve (AUC) of the receiver operator characteristic (ROC)^17^ was calculated to assess predictive power of EGFR-sensitizing mutation. Since only 13 features were tested, correction for multiple testing was not considered. All statistical analysis was conducted using the R statistical software version 3.1.0^18^ on a Linux operating system.

### Technical validation

Technical validation of the features were conducted in a test-retest setting on the RIDER dataset, which contains of 31 patients each two lung CT scans taken approximately 15 min. apart. The RIDER lung cancer dataset^19^ of the same-day repeat CT scans and the intraclass correlation coefficient (ICC) was used to assess the stability of features for test-retest. The CT imaging protocol of the RIDER data set was identical to the one used in this study^20^.

## Results

**Figure 1:**
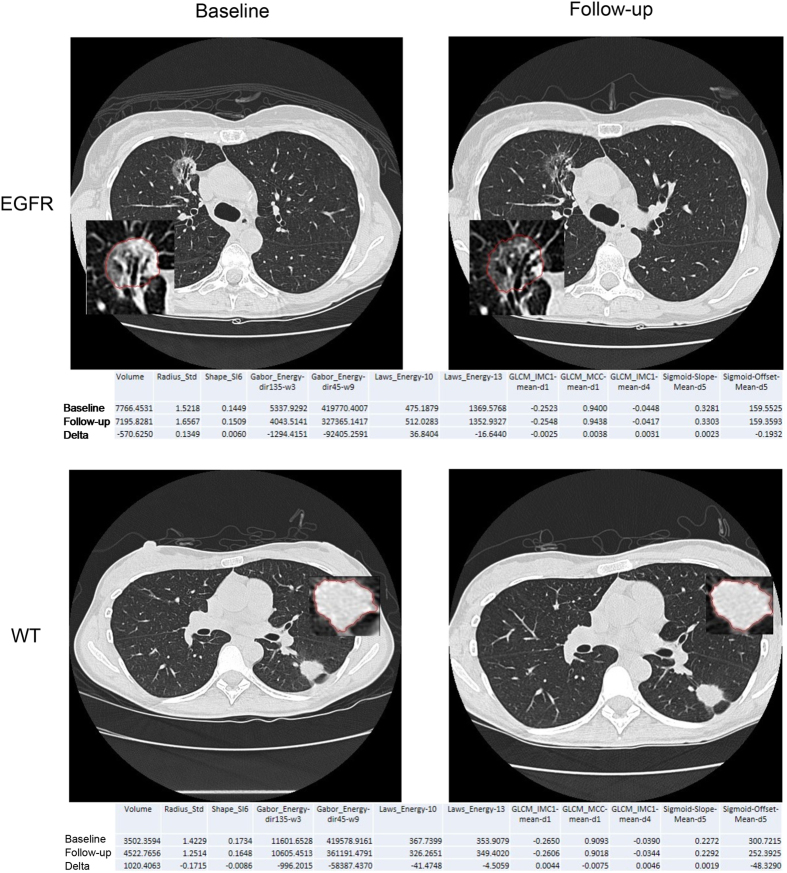
Example images of a patient with EGFR mutation and without (wild-type; WT) at baseline and follow-up scan. Radiomic feature values are given below each image for baseline and follow-up time points, as well as their delta differences.

**Figure 2:**
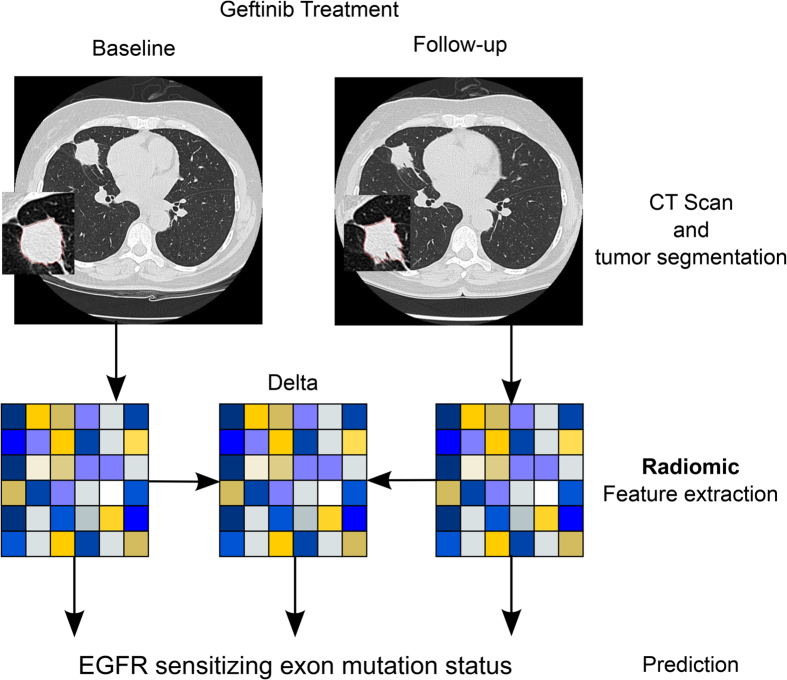
Study Design. Patients included to the study were received Gefitinib treatment. CT scans at baseline and first follow-up were used to segment the tumor and to extract radiomic features. Baseline, follow-up, and delta radiomics (differences between baseline and follow-up) were used to assess EGFR sensitizing mutation status.

To investigate if radiomic biomarkers are associated with mutational status and response to Gefitinib treatment, we analyzed clinical data of 47 early stage NSCLC patients whom were imaged before and after treatment. In Fig. 1 representative CT scans of an EGFR mutant and an EGFR wild-type tumor are shown before and after Gefitinib treatment, demonstrating clear phenotypic differences. To quantify these differences, we performed a radiomic analysis (see Fig. 2). The analysis was restricted to features with high and independence variance (Supplement I), resulting in eleven radiomic features and two volumetric features (volume and max diameter), that were included in our analysis (see Table 1). Using this strategy, we were able to identify a limited number of independent features, and only these features were assessed for performance to predict mutational status and associated Gefitinib response.

### Baseline radiomics associations with mutational status

**Figure 3:**
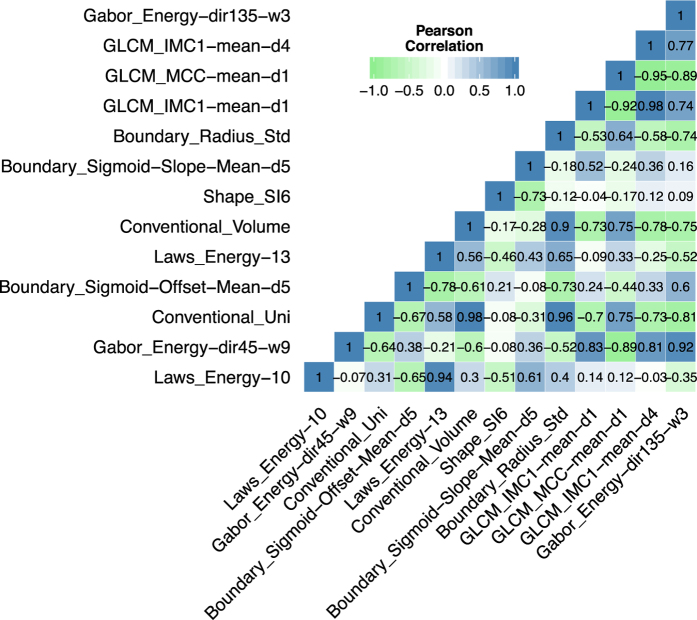
Correlations of Radiomics features. Correlation coefficient matrix between the 13 imaging features evaluated in the analysis. Note the overall low correlation between radiomic features. Correlations were assessed using pearson correlation coefficient.

We investigated the correlations between the image features evaluated in our analysis. In Fig. 3 the correlations between the features extracted from the before treatment CT scan are shown. Although the GLCM features showed high positive and negative correlations, overall the correlations between those features were low (mean ± std: −0.16 ± 0.95), demonstrating independency of those features. Note the low correlation of total tumor volume with the other features (mean ± std: 0.01± 0.33).

**Figure 4:**
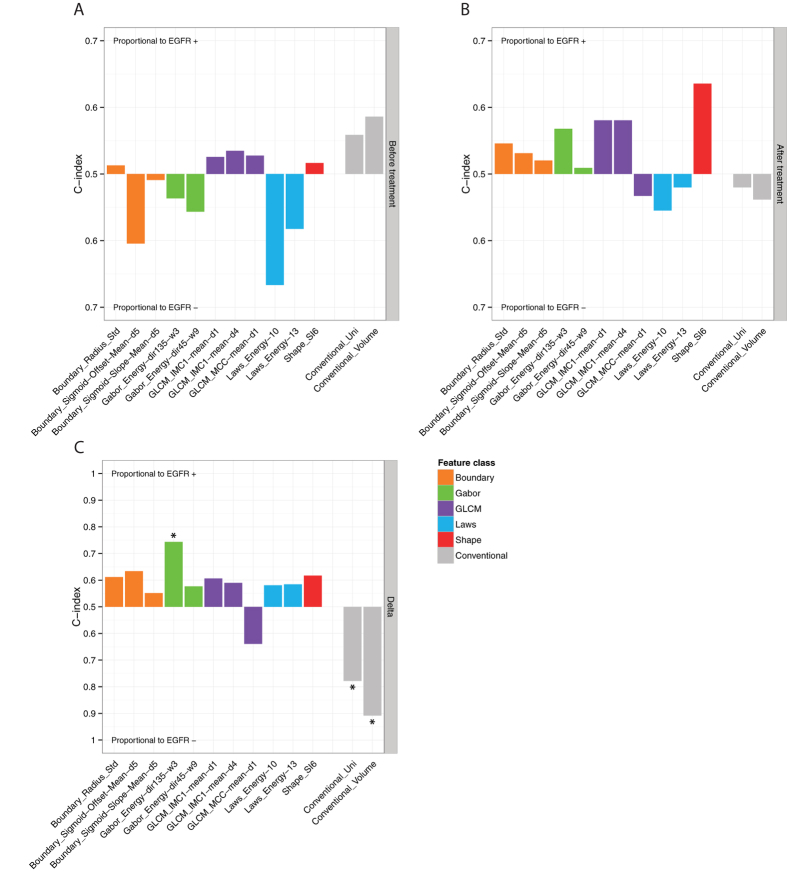
Value of radiomic features to predict mutational status extracted from (**A**) before treatment scan, (**B**) post-treatment scan, and (**C**) delta (difference between the scan before and after treatment). Predictive value is assessed using the area under the curve (AUC) of the receiver operator characteristic (ROC). Asterisk denotes features that significantly predict mutation status better than random (*p* < 0.05). Note, before treatment only the radiomic feature Laws_Energy was significant for predicting mutation status, and conventional imaging markers showed no significant predictive power. After treatment no features are predictive, however delta feature values between the pre and post-scan show strongest predictive value.

To assess the associations of radiomic features and EGFR mutational status, we evaluated the predictive power using the AUC of the ROC. Figure 4 shows the AUC values that were measured for imaging features extracted from the scan before treatment, after treatment, and the difference between both scans (delta). Interestingly, Laws Energy 10 is the only radiomic feature that is significantly predictive for mutational status extracted from the pre-treatment scan (AUC = 0.67, p = 0.03). Note that pre-treatment tumor volume (AUC = 0.59, p = 0.27) and maximum diameter (AUC = 0.56, p = 0.46) are not predictive of mutation status. No features are significantly predictive extracted from the image scan after treatment (highest AUC = 0.64, p = 0.08, Shape SI6). Also, the remaining volume after treatment is not predictive (AUC = 0.54, p = 0.63).

### Response phenotyping by radiomics feature change to predict mutational status

To assess the difference in radiomic feature values between the two scans (delta), showed strong predictability for mutation status (Fig. 4C). The strongest predictors are delta volume (AUC = 0.91, p = 10^−25^) and delta maximum diameter (AUC = 0.78, p = 10^−5^). However, one radiomic feature was also significantly predictive: delta Gabor Energy (dir135-w3), which is Gabor Energy calculated at the wavelength of 3 pixels and direction of 135 grade (AUC = 0.74, p = 3 *×* 10^−4^). Although, this feature is predictive, the correlations with tumor volume (*r* = −0.31) and maximum diameter (*r* = −0.43) are low (Fig. 3). These results demonstrate the predictability of radiomic features quantifying phenotypic characteristics other than volumetric features.

### Technical validation of radiomic features for stability

As a technical validation, we assessed the stability of the four features that showed significantly predictive capabilities on either the pre, after, or delta scans, i.e. (I) Laws Energy, (II) Gabor Energy, (III) tumor volume, and (IV) maximum diameter (Fig. 4). For this purpose we used the independent RIDER lung cancer dataset consists of same-day repeated CT scans for 31 patients. The intraclass correlation coefficient (ICC) was used to assess the stability of each feature for test-retest. We found that all four significantly associated features with mutation status were also highly stable for test-retest (mean ± std: ICC = 0.96 ± 0.06). Feature Laws_Energy_10, the only feature significantly associated with mutational status extracted from the pretreatment scan, had a high stability of ICC = 0.87. The stability of the features significantly associated with mutational status between the two time points, was also very high: Volume (ICC = 0.99), Gabo Energy (ICC = 0.97), and maximum diameter (ICC = 0.99). These results validate that features significantly associated with mutation status are also stable for test-retest.

## Discussion

Biomarkers that are able to predict treatment response are crucial for clinical introduction of targeted therapies. Erlotinib and Gefitinib are examples of targeted therapies that were successfully introduced in practice, largely due to accurate predictive biomarkers (i.e. EGFR mutation status). Others have failed to become clinically approved, such as Cetuximab in NSCLC, to a great extent because an accurate response biomarker is lacking^21^,^22^,^23^,^24^. Medical imaging is intuitively very suitable for this purpose, as it is able to visualize and quantify time series of disease processes in a non-invasive way in individual patients. “Radiomics”, the extraction and analysis of large amounts of advanced imaging features, is able to quantify tumor phenotypic properties, thereby potentially providing valuable diagnostic, prognostic or predictive information. Radiomic features have been associated with clinical outcomes^4^,^8^,^25^,^26^,^27^,^28^,^29^, however the predictive capability of radiomics for response to targeted therapies remains largely unknown.

The goal of this study was to investigate if radiomic data could define a response phenotype for NSCLC patients treated with Gefitinib therapy. Our data show strong associations between radiomic features and tumors with and without sensitizing mutations. The radiomics feature Laws Energy extracted from the baseline, pretreatment CT scan showed the strongest performance to predict mutation status (AUC = 0.67, p = 0.03). In contrast, we found that tumor volume and maximum diameter were both not significantly predictive (p > 0.27). These data show that radiomics analyses can reveal more phenotypic characteristics than standard imaging features, such as size and volume, and have tremendous potential to be incorporated into precise response biomarkers. However, before clinical applicability, the predictive performance of these features have to be evaluated in large independent cohorts across institutions.

As expected, response of imaging phenotype was extremely different for patients with and without sensitizing mutations. We compared the radiomic data extracted from the change in pre- and post-treatment scans. As expected, tumor volume and maximum diameter had the highest performance; however the radiomic feature Gabor Energy was also significantly predictive. The correlation of this feature with tumor volume and maximum diameter was low. This could indicate the independent predictability for mutational status; however, future studies are needed to show if these features have complementary value in multivariate models for response prediction or may be seen earlier in treatment response. Due to the limited sample size of this study, we were not able to identify and validate multivariate biomarkers. The analysis supports CT-based response assessment as one effective tool for distinguishing sensitive and resistant tumor, but there may be other metrics that are additive and would allow a more comprehensive measure of response. The specific radiomic features may depend upon the specific mechanism of action of the drug or class of therapy.

Correlations between our selected radiomics features demonstrated that in general, the features originally derived from CT segmentation of tumors are indeed independent measures of phenotypic characteristics. Each feature has the potential to offer unique insight to the tumor behavior. This was demonstrated by the fact that different radiomic features predicted for tumors with and without EGFR mutations, and confirmed by the relative independence between most of the imaging features. However, future studies have to investigate how these patterns in the imaging data captured by these radiomic features, are associated with the underlying driving biology.

As a technical validation we assessed if the four features significantly associated with mutation status, identified by our analysis, could be stably extracted from CT scans. We evaluated this stability using another dataset with repeated test-retest CT scans for 31 patients (RIDER). All four features could be validated and had high stability for test-retest (ICC > 0.87).

Variability in CT acquisition and reconstruction parameters is inherent in clinical practice. In this study we used a prospective collected dataset with high resolution CT scans with the same imaging protocol for all included patients. However, the optimal scanning parameters still have to be defined and more domain specific quantitative features are anticipated to be developed. The Quantitative Imaging Network (QIN) of the National Institute of Health, and others, play an important role in this process by performing phantom studies, radiomic feature definition standardizations, and segmentation challenges^30^. Furthermore, they establishes open and standardized protocols for image acquisition, reconstruction, and analysis^30^,^31^,^32^. It is expected that these efforts will improve the predictive performance of radiomic response biomarkers even further.

As this was a pilot investigation our analysis had several limitations. A main limitation was the limited sample size as we included only 47 patients in our analysis. As the data was acquired in a research study, with high quality imaging data and imaging before and after gefinitib treatment, more data was not available. Also, we limited our analysis to eleven independent radiomic features. A full radiomic analysis^4^, evaluating hundreds of features, could potentially achieve higher performance radiomic biomarkers for gefitinib response, however requires large independent training and validation cohorts. Furthermore, before clinical application imaging protocols have to be standardized and hence variability in CT acquisition and reconstruction parameters in clinical practice has to be reduced. The Quantitative Imaging Biomarker Alliance from the Radiological Society of North America (RSNA) and the QIN play an important role in this process by establishing standards for image acquisition and reconstruction, by conducting phantom studies, and by performing segmentation challenges. In addition, multiples studies have already documented the robustness of radiomic feature extractions in terms of reproducibility and repeatability in test/re-test settings^4^,^19^,^27^. However, before clinical utility, future studies have to evaluate radiomic biomarkers in independent and prospective validation cohorts with large sample sizes, and show improved performance compared to volumetric imaging features.

In conclusion, we found that radiomics features are able to define a Gefitinib response phenotype non-invasively, that is able to distinguish between tumors with and without EGFR sensitizing mutations at baseline and a quantitative change in these radiomic features at follow up. The use of radiomics based response assessment tools could improve the stratification between sensitive and resistant patient populations and the detection of response to treatment. This may provide an opportunity to improve decision-support at low aditional cost, as imaging is routinely and repeatedly used in clinical practice.

## Additional Information

**How to cite this article**: Aerts, H. J. W. L. *et al.* Defining a Radiomic Response Phenotype: A Pilot Study using targeted therapy in NSCLC. *Sci. Rep.* **6**, 33860; doi: 10.1038/srep33860 (2016).

## Supplementary Material

Supplementary Information

## Figures and Tables

**Table 1:** Definitions of evaluated quantitative image features.

Feature	Description
**Volume**	Tumor volume is calculated by multiplying the number of tumor voxels by the image resolutions in x-, y- and z-directions.
Gabor Energy feature class	Gabor filters are linear filters designed for edge detection. This is an oriented Gaussian function modulated by a sinusoidal wave. The Gabor Energy feature is defined as the sum of the square of density over all lesion pixels on the images pre-processed by Gabor filter.· Gabor_Energy-dir135-w3: the Gabor Energy feature calculated on the images pre-processed using the Gabor filter built with an orientation of 135° and a wavelength of 3 pixels.· Gabor_Energy-dir45-w9: the Gabor Energy feature calculated on the images pre-processed using the Gabor filter built with an orientation of 45° and a wavelength of 9 pixels.
Sigmoid Function feature class	To quantify lesion margins, Sigmoid curve is used to fit density change along a sampling line drawn orthogonal to the lesion surface. Each sampling line, going through one voxel on the lesion surface, has a certain length inside and outside the lesion.· Sigmoid-Offet-Mean-d5: The average of the densities between a lesion and lung parenchyma on all lines. The line length is 5 mm at both sides of the lesion.· Sigmoid-Slope-Mean-d5: The average of the density change speed between lesion and lung parenchyma on all lines. The line length is 5 mm at both sides of the lesion.
Shape Index feature class	Local surface shape of a 3D object can be intuitively captured by 9 Shape Index features, Shape_SI1-9. The value of each Shape Index ranges from 0 to 1. The larger the value, the greater the portion of the shape on the surface.· Shape_SI6: Describes the saddle ridge shape.
Boundary_Radius_Std	This feature is defined as the standard deviation of the lengths of the line segments from the center of an object to any voxel on the surface of the object. A spherical shape has the smallest value of zero (0).
GLCM feature class	GLCM stands for grey-level co-occurrence matrix. This feature class characterizes image textures by creating a new matrix, GLCM, which is based on the frequency of image pixel pairs having particular gray-level values in a particular spatial arrangement (i.e., distance and direction). A number of statistical features can then be extracted from GLCM to characterize homogeneity, contrast, entropy and so on. In the following feature names, “mean” specifies average of feature values calculated at 13 directions. “d1” and “d4” indicate pixel pairs separated by 1-pixel distance and 4-pixel distance.· GLCM_IMC1-mean-d1: The average of informational measure of correlation 1 calculated at pixel pairs separated by 1-pixel distance· GLCM_IMC1-mean-d4: The average of informational measure of correlation 1 calculated at pixel pairs separated by 4-pixel distance· GLCM_MCC-mean-d1: The average of Maximum Correlation Coefficient calculated at pixel pairs separated by 1-pixel distance. calculated at pixel pairs separated by 4-pixel distance.
Laws Energy feature class	Laws’ Energy emphasizes edge, spot, ripple and wave patterns through Laws filters generated by the following 5 basic raw vectors: Average *L*_5_ = (1, 4, 6, 4, 1), Edge *E*_5_ = (−1, −2, 0, 2, 1), Spot *S*_5_ = (−1, 0, 2, 0, −1), Ripple *R*_5_ = (1, −4, 6, −4, 1), and Wave. By multiplying and combining the transpose of one basic vector and/or the vector itself, 14 standard Laws filters can be built, each generating one feature. A Laws Energy feature is computed by summing the square of image pixel value over all tumor pixels on images processed by one of the 14 Laws filters.
	• Laws_Energy-10: Energy calculated on the images processed by Laws filter #10 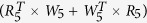 . • Laws_Energy-13: Energy calculated on the images processed by Laws filter #13 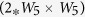 .
