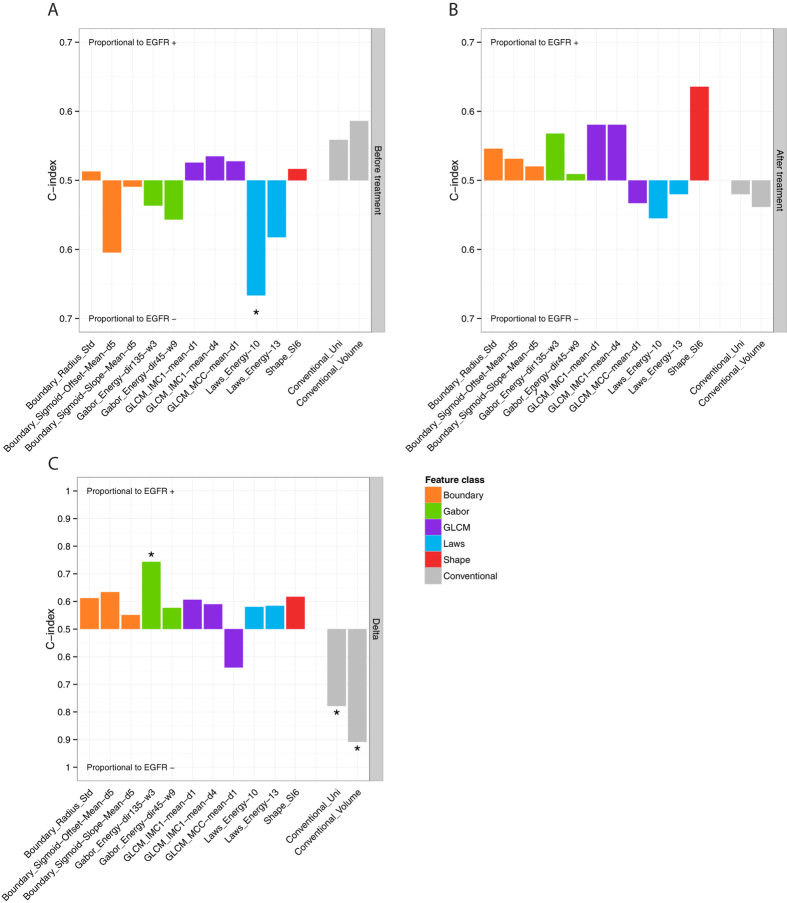# Corrigendum: Defining a Radiomic Response Phenotype: A Pilot Study using targeted therapy in NSCLC

**DOI:** 10.1038/srep41197

**Published:** 2017-02-17

**Authors:** Hugo J. W. L. Aerts, Patrick Grossmann, Yongqiang Tan, Geoffrey R. Oxnard, Naiyer Rizvi, Lawrence H. Schwartz, Binsheng Zhao

Scientific Reports 6: Article number: 3386010.1038/srep33860; published online: 09 20 2016; updated: 02 17 2017

The original version of this Article contained a typographical error in the spelling of the author Geoffrey R. Oxnard, which was incorrectly given as Geoffrey G. Oxnard.

Additionally, the original version of this Article contained an error in Figure 4A, where an asterisk was omitted in error. The correct Figure 4 appears below as Figure 1.

These errors have now been corrected in the PDF and HTML versions of the Article.

## Figures and Tables

**Figure 1:**