# Supplementary material for: Defining a Radiomic Response Phenotype: A Pilot Study using targeted therapy in NSCLC
Source: Sci Rep. 2016 Sep 20;6:33860. doi: 10.1038/srep33860 (PMC5028716; doi:10.1038/srep33860)
Supplement: Supplementary Information [file srep33860-s1.pdf]

Supplement I

Defining a Radiomic Response Phenotype: A Pilot Study using targeted therapy in NSCLC

Hugo J.W.L. Aerts[1,2,3,*], Patrick Grossmann[1,3,*], Yongqiang Tan[4], Geoffrey G Oxnard[6], Naiyer Rizvi[5], Lawrence H. Schwartz[4], Binsheng Zhao[4]

Departments of [1]Radiation Oncology and [2]Radiology, [3]Biostatistics & Computational Biology Dana-Farber Cancer Institute, Brigham and Women's Hospital, Harvard Medical School, Boston, MA, USA, [4]Department of Radiology Columbia University College of Physicians and Surgeons and New York Presbyterian Hospital, New York, NY, USA, Department of Medicine, [5]Division of Oncology Columbia University College of Physicians and Surgeons and New York Presbyterian Hospital, New York, NY, USA, [6]Lowe Center for Thoracic Oncology, Dana-Farber Cancer Institute, Boston, MA, * equal contribution

Tables

In this work, 183 quantitative image features are extracted, some in 2D, some in 2.5D and some in 3D. 2D features are computed on the image where the unidimensional measurement of the tumor is calculated. A 2.5D feature is defined as the average of the features computed on all single images containing the tumor. As to the numbers of neighboring pixels (2D) / voxels (3D), 8 connected pixels are considered for 2D analysis, whereas 26 connected voxels are considered for 3D analysis. 8 and 13 directions are used for 2D and 3D analysis, respectively.

Feature Definitions

No.	Feature / Dimension	Description
1	Uni / 1D	Maximal diameter of tumor. Calculated by multiplying the longest line inside the tumor on an axial plane by the image resolution in x or y direction.
2	Volume / 3D	Volume of tumor. Calculated by multiplying the number of tumor voxels by the image resolutions in x-, y- and z-directions.
3-12	First Order Statistics / 2D and 3D	Features in this group are derived from the intensity histogram of tumor. The following 5 well-known first order statistics are calculated in both 3D and 2D, totaling 10 features in this group.<br>• Mean: average intensity of tumor<br>• SD: standard deviation of tumor intensities<br>• Skewness: a measure of intensity symmetry<br>• Kurtosis: a measure of flatness of tumor intensity relative to a normal distribution<br>• Peak Position: the peak position of the histogram
13-21	Shape / 2D and 3D	This group of features describes the shape properties of the tumor.<br>In 2D, the following three shape features are extracted:<br>• Roundness Factor (*RF*): a measure of circularity of a tumor's profile on the 2D image. It is defined as, $RF = \frac{4\pi \cdot Area}{Perimeter^{2}}$ where *Area* denote the area of tumor and *Perimeter* the length of tumor contour on the 2D image.<br>• Eccentricity: a measure specifying how close an ellipse (i.e., a tumor's profile on the 2D image) is to a circle. It is defined as:

		Eccentricity = c / a<br>where, *c* is the distance from the center to a focus and *a* is the distance from that focus to a vertex.<br>• Solidity: a measure of convexity of a tumor's profile on the 2D image. It is defined as,<br>$Solidity = \frac{Area}{ConvexArea}$<br>where *Area* denote the tumor's area and *ConvexArea* the area of the convex hull bounding the tumor on the 2D image.<br>In 3D, the following 6 shape features are extracted:<br>• Compactness Factor (*CF*): a measure of sphericity of tumor in 3D. It is defined as,<br>$CF = \frac{V}{\sqrt{\pi}S^{\frac{2}{3}}}$<br>where, V denotes tumor volume and S tumor surface.<br>• Ratio of minor to major axes: the ratio of tumor shortest diameter to its longest diameter in 3D<br>• Ratio of minor to middle axis: the ratio of tumor shortest diameter to its perpendicular, second longest diameter perpendicular to in 3D<br>• Ratio of tumor volume to its surrounding box volume: a measure to quantify tumor's convexity in 3D. The surrounding box is defined as the smallest sphere encompassing the tumor<br>• Mean of radii: the mean of the lengths of the line segments from the center of tumor to any voxel on the surface of tumor<br>• Standard Deviation of radii: the standard deviation of the lengths of the line segments from the center of tumor to any voxel on the surface of tumor
22-29	Surface Shape / 3D	Shape index is used to intuitively characterize the local shapes of tumor surface [1]. The shape indexes, SI(1) – SI(9), are numbers between [-1, 1] and respectively imply the amount of the following 9 shapes on tumor surface:<br>• SI(1) $\in [-1, -\frac{7}{8})$: spherical cup<br>• SI(2) $\in [-\frac{7}{8}, -\frac{5}{8})$: trough<br>• SI(3) $\in [-\frac{5}{8}, -\frac{3}{8})$: rut<br>• SI(4) $\in [-\frac{3}{8}, -\frac{1}{8})$: saddle rut<br>• SI(5) $\in [-\frac{1}{8}, \frac{1}{8})$: saddle<br>• SI(6) $\in [\frac{1}{8}, \frac{3}{8})$: saddle ridge

		• SI(7) ∈ $[\frac{3}{8}, \frac{5}{8})$: ridge<br>• SI(8) ∈ $[\frac{5}{8}, \frac{7}{8})$: dome<br>• SI(9) ∈ $[\frac{7}{8}, 1]$: spherical cap<br>Since the sum of the 9 scaled shape indexes equals to 1, SI(1) is excluded from the analysis to reduce the redundancy. This group has 8 features.
30-35	Sigmoid Function / 3D	To quantify tumor margins, Sigmoid Function is used to fit density change along a sampling line drawn orthogonal to the tumor surface [2]. Each sampling line, going through one voxel on the tumor surface, has a certain length (5mm in this work) inside and outside the tumor. The Sigmoid Function is defined as,<br>$Sigmoid(x) = \frac{A}{e^{B \cdot x} + 1} + C$<br>where the fitting parameter *A*, *B* and *C* respectively specify the amplitude, slope and offset of the curve. The 6 Sigmoid Function features in this group are as follows:<br>• Sigmoid_Amplitude_Mean: average of the amplitude values (A) of all sampling lines<br>• Sigmoid_Amplitude_SD: Standard deviation of the amplitude values (A) of all sampling lines<br>• Sigmoid_Slope_Mean: average of the slope values (B) of all sampling lines<br>• Sigmoid_Slope_SD: Standard deviation of the slope values (B) of all sampling lines<br>• Sigmoid_Offset_Mean: average of the offset values (C) of all sampling lines<br>• Sigmoid_Offset_SD: Standard deviation of the offset values (C) of all sampling lines
36-53	Wavelets / 2.5D	In this study, two types of Wavelets transform are implemented. One is discrete wavelet transform (DWT)[3] and the other is discrete stationary wavelet transform (SWT) [4]. For each transform, Wavelets images/features are calculated at three decomposition levels, resulting in 18 features in this group.<br>DWT: Taking a M x N image $I(m, n)$ as an example, the first level DWT decomposition can be briefly described as the following. First, a low-pass and a high-pass filter ('Coiflets1' wavelet filter) are applied to the original image vertically followed with a vertical down-sampling by a factor of 2. Then the two filters are applied to the processed image horizontally followed by a horizontal down-sampling by a factor of 2. This results in 4 sub-images that are known as the low-pass approximation $L(m, n)$ (average image), vertical detail $V(m,n)$,

		horizontal detail *H*(*m, n*) and diagonal detail *D*(*m, n*). The second (and (third) level DWT decomposition repeats the above procedure but with the average image generated at the first (and second) level decomposition.<br><br>In this study, 9 wavelet features are defined as the Energy of each detailed sub-images. Let N_i be the number of pixels of a sub-image at level *i* (*i*=1, 2).<br><br>At the first DWT decomposition level,<br><br>• DWT-H:<br>$Energy_H = \sum_i^N H(i)^2$<br>• DWT-V:<br>$Energy_V = \sum_i^N V(i)^2$<br>• DWT-D:<br>$Energy_D = \sum_i^N D(i)^2$<br><br>At the second DWT decomposition level,<br>• DWT-LH:<br>$Energy_{LH} = \sum_i^N LH(i)^2$<br>• DWT-LV:<br>$Energy_{LV} = \sum_i^N LV(i)^2$<br>• DWT-LD:<br>$Energy_{LD} = \sum_i^N LD(i)^2$<br><br>At the third DWT decomposition level,<br>• DWT-LH:<br>$Energy_{LLH} = \sum_i^N LLH(i)^2$<br>• DWT-LV:<br>$Energy_{LLV} = \sum_i^N LLV(i)^2$

		• DWT-LD: $$Energy_{LLD} = \sum_{i}^{N} LLD(i)^2$$ SWT:<br>To overcome the translation-variance in DWT, SWT performs up-sampling on wavelet filters instead of down-sampling on the image. Thus, at each scale of SWT decomposition, the image is convolved by up-sampled filter coefficients and remains the original size. There are seven corresponding SWT features (detail not included).
54-62	Edge Frequency / 2.5D	Edge Frequency features, obtained from images processed by an edge operator (in this work, it is a 2D Robert's edge operator), characterize variation of the intensity gradient inside a tumor [5]. The 2D Robert's edge operator is defined as follows: $$gradient(d) = \lvert f(i,j) - f(i+d,j)\rvert + \lvert f(i,j) - f(i-d,j)\rvert + \lvert f(i,j) - f(i,j+d)\rvert + \lvert f(i,j) - f(i,j-d)\rvert$$ where $f(i,j)$ denotes a tumor pixel intensity at the location (i, j), and d is the distance between the pixel $f(i,j)$ and its neighboring pixel. The three features, Mean, Coarseness and Contrast, are computed from the processed gradient images at the distances of one, four and nine pixels, which totals 9 features. Equations to define the Coarseness and Contrast are the same as the ones defined in the GTDM feature group, with $S(i)$ replaced by gradient(d).
63-64	Fractal Dimension / 2.5D	Fractal Dimension provides a statistical index to characterize the complexity of an image [6]. Basically, the Fractal Dimension describes the relationship between the changes in a measuring scale and the measurement results at the scale. In this work, a 3D box-counting algorithm [7] is adopted to calculate the Fractal Dimension to quantify tumor intensity homogeneity. Fractal Dimension Mean and Standard Deviation are computed.
65-79	Gray-Tone Difference Matrix (GTDM) / 2.5D	Neighborhood GDTM features are defined based on gray-tone (i.e., image intensity) difference between a pixel and its neighborhood [8]. Let $f(k,l)$ be an image pixel that has the gray-tone of i and is located at (k,l). The average gray-tone over a neighborhood centered at, but excluding (k,l), is

$$\bar{A}_i = \bar{A}(k,l) = \frac{1}{W-1}\left[\sum_{m=-d}^{d}\sum_{n=-d}^{d} f(k+m, l+n)\right] \quad (m,n) \neq (0,0)$$

where $W = (2d+1)^2$ is the neighborhood size (area). The ith entry in the GDTM is

$$s(i) = \sum\left|i - \bar{A}_i\right|, \quad \text{for } i \in N_i \text{ if } N_i \neq 0, \\ = 0, \quad \text{otherwise}$$

where $\{N_i\}$ is the set of all pixels having the gray-tone of i. For an $N \times N$ image, let p_i be the probability of occurrence of gray-tone value i, L_h be the highest gray-tone value in the image and N_g be the total number of different gray-tone values in the image. The GDTM features are defined as,

- Coarseness:

$$Coarseness = \left[\sum_{i=0}^{L_h} p_i s(i)\right]^{-1}$$

- Contrast:

$$Contrast = \left[\frac{1}{N_g(N_g-1)}\sum_{i=0}^{L_h}\sum_{j=0}^{L_h} p_i p_j (i-j)^2\right]\left[\frac{1}{n^2}\sum_{i=0}^{L_h} s(i)\right]$$

- Busyness:

$$Busyness = \frac{\left[\sum_{i=0}^{L_h} p_i s(i)\right]}{\left[\sum_{i=0}^{L_h}\sum_{j=0}^{L_h}(ip_i - jp_j)\right]}, p_i \neq 0, p_j \neq 0$$

- Complexity:

		$$Complexity = \sum_{i=0}^{L_h}\sum_{j=0}^{L_h}\{(\lvert i-j\rvert)/\left(n^2(p_i+p_j)\right)\}\{p_i s(i)+p_j s(j)\}, p_i \neq 0, p_j \neq 0$$<br>• Strength:<br>$$Strenght = \frac{\left[\sum_{i=0}^{L_h}\sum_{j=0}^{L_h}(p_i+p_j)(i-j)^2\right]}{\left[\sum_{i=0}^{L_h} s(i)\right]}, p_i \neq 0, p_j \neq 0$$<br>In this study, $N_g = 256$. The distance of the neighboring pixels is 1.
80-99	Gabor / 2.5D	Gabor filters are linear filters designed for detecting edges at different directions and width [9]. It is an oriented Gaussian function modulated by a sinusoidal wave. The Gabor Energy feature is defined as the sum of the square of intensity over all tumor pixels on the images processed by Gabor filter. In this work, the Energy is calculated at 4 wavelengths (w=3, 5, 7 and 9 pixels) and at each wavelength, there are 4 directions and the sum of all directions. This totals up to 20 Gabor features. Below are 5 example Gabor features computed at 4 directions (dir=0,45, 90 and 135) and the sum of all directions, with the wavelength of 3 pixels.<br>• Gabor_Energy-dir0-w3: the Energy feature calculated on images processed with the Gabor filter built with an orientation of 0° and wavelength of 3 pixels<br>• Gabor_Energy-dir45-w3: the Energy feature calculated on images processed with the Gabor filter built with an orientation of 45° and wavelength of 3 pixels<br>• Gabor_Energy-dir90-w3: the Energy feature calculated on images processed with the Gabor filter built with anorientation of 90° and wavelength of 3 pixels<br>• Gabor_Energy-dir135-w3: the Energy feature calculated on images processed with the Gabor filter built with an orientation of 135° and wavelength of 3 pixels<br>• Gabor_Energy-sum-w3: the Gabor Energy feature calculated on images processed using the Gabor filter built with the sum of all of the above 4 orientation at

		the wavelength of 3 pixels
100-113	Laws Energy / 2.5D	Laws' Energy emphasizes edge, spot, ripple and wave patterns through Laws filters generated by the following 5 basic raw vectors: Average $L_5 = (1,4,6,4,1)$, Edge $E_5 = (-1,-2,0,2,1)$, Spot $S_5 = (-1,0,2,0,-1)$, Ripple $R_5 = (1,-4,6,-4,1)$, and Wave $W_5 = (-1,2,0,-2,-1)$[10]. By multiplying and combining the transpose of one basic vector and/or the vector itself, 14 standard Laws filters can be built, each generating one feature. A Laws Energy feature is computed by summing the square of image pixel value over all tumor pixels on images processed by one of the 14 Laws filters.<br>• Laws_Energy-1: Energy calculated on the images processed by Laws filter #1 ($E_5^T \times L_5 + L_5^T \times E_5$)<br>• Laws_Energy-2: Energy calculated on the images processed by Laws filter #2 ($S_5^T \times L_5 + L_5^T \times S_5$)<br>• Laws_Energy-3: Energy calculated on the images processed by Laws filter #3 ($W_5^T \times L_5 + L_5^T \times W_5$)<br>• Laws_Energy-4: Energy calculated on the images processed by Laws filter #4 ($R_5^T \times L_5 + L_5^T \times R_5$)<br>• Laws_Energy-5: Energy calculated on the images processed by Laws filter #5 ($S_5^T \times E_5 + E_5^T \times S_5$)<br>• Laws_Energy-6: Energy calculated on the images processed by Laws filter #6 ($W_5^T \times E_5 + E_5^T \times W_5$).<br>• Laws_Energy-7: Energy calculated on the images processed by Laws filter #7 ($R_5^T \times E_5 + E_5^T \times R_5$)<br>• Laws_Energy-8: Energy calculated on the images processed by Laws filter #8 ($W_5^T \times S_5 + S_5^T \times W_5$)<br>• Laws_Energy-9: Energy calculated on the images processed by Laws filter #9 ($R_5^T \times S_5 + S_5^T \times R_5$)<br>• Laws_Energy-10: Energy calculated on the images processed by Laws filter #10 ($R_5^T \times W_5 + W_5^T \times R_5$)<br>• Laws_Energy-11: Energy calculated on the images processed by Laws filter #11 ($2 * E_5 \times E_5$)<br>• Laws_Energy-12: Energy calculated on the images processed by Laws filter #12 ($2 * S_5 \times S_5$)<br>• Laws_Energy-13: Energy calculated on the images processed by Laws filter #13 ($2 * W_5 \times W_5$)<br>• Laws_Energy-14: Energy calculated on the images processed by Laws filter #14 ($2 * R_5 \times R_5$)
114-125	LoG / 2.5D	Laplacian of Gaussian (LoG) is a combined filter, i.e., a Gaussian smoothing filter followed by Laplacian, a differential operator [11]. The definition of a 2D LoG is:<br>$$\mathrm{LoG}(x,y) = -\frac{1}{\pi\sigma^4}\left[1-\frac{x^2+y^2}{2\sigma^2}\right]e^{-\frac{x^2+y^2}{2\sigma^2}},$$

		In this study, the following three LoG features of Mean, Uniformity and Entropy are calculated from the LoG filtered (processed) image, $LoGMask(i)$, at four σ levels $\sigma \in [0, 0.5, 1.5, 2.5]$. $\sigma = 0$ (s1; no smoothing) and $\sigma = 2.5$ (s4). This totals 12 LoG features in this group.<br><br>• LoG Mean Gray Intensity (MGI):<br>$$Mean = \sum_{\mathrm{i}}^{\mathrm{N}} LoGMask(i)^2$$<br>• LoG Uniformity:<br>$$Uniformity = \sum_{i=1}^{L} P(i)^2$$<br>• LoG entropy:<br>$$Entropy = -\sum_{i=1}^{L} P(i)\log_2 P(i)$$<br>where N is the number of object pixels, P(i) is the probability of pixels with a gray-level of i in the LoG pre-processed image, and L is the maximal value of the pre-processed image.
126-130	Run-length / 3D	The Run-length features are developed to characterize tumor heterogeneity by counting the number of maximum contiguous pixels / voxels having an identical intensity along a line [12]. Let $R(a,r)$ be the number of primitives of all directions having length of r and intensity of a, V the volume of tumor, N_r the maximum run-length, and $L = 256$ the number of image intensity bins. The total number of run-lengths is therefore,<br>$$K = \sum_{a=1}^{L}\sum_{r=1}^{N_r} R(a,r)$$<br>In this work, the following five Run-length features are used.<br>• Run_SPE: Short primitives emphasis<br>$SPE = \frac{1}{K}\sum_{a=1}^{L}\sum_{r=1}^{N_r}\frac{R(a,r)}{r^2}$<br>• Run_LPE: Long primitives emphasis<br>$$LPE = \frac{1}{K}\sum_{a=1}^{L}\sum_{r=1}^{N_r} R(a,r)r^2$$<br>• Run_GLU: Gray-level uniformity<br>$$GLU = \frac{1}{K}\sum_{a=1}^{L}\left[\sum_{r=1}^{N_r} R(a,r)\right]^2$$

		• Run_PLU: Primitive length uniformity<br>$$PLU = \frac{1}{K}\sum_{r=1}^{N_r}\left[\sum_{a=1}^{L} R(a,r)\right]^2$$<br>• Run_PP: Primitive percentage<br>$$PP = \frac{K}{V}$$
131-132	Spatial Correlation / 2.5D	Spatial Correlation features assess linear spatial relationships between texture primitives [13]. Let $I(i,j)$ be an image pixel's intensity at the location (x, y) in a tumor, d the distance between two pixels, S the area of the tumor, S_d the area of the tumor after shrinking with a distance of d pixels. Then,<br>$$Spatial\ Correlation = \frac{S}{S_d}\frac{\sum_{i,j=1}^{S_d} I(i,j)I(i+d,j+d)}{\sum_{i,j=1}^{S} I(i,j)^2}$$<br>In this study, the spatial correlations are calculated at the distances of d=1 and d=4 pixels.
133-183	Gray-Level Co-occurrence Matrix (GLCM) /3D	This feature class quantifies textures by creating a new matrix, called GLCM, which is based on the frequency of image pixel pairs possessing particular intensity values at a certain direction and distance [14]. In this work, GLCMs are generated at 13 directions and three distances (d=1, 4, 9 voxels). At each distance, the final GLCM is the average of the 13 GLCMs. For each of the three GLCMs, the following 17 standard statistical features are derived to characterize tumor's homogeneity, contrast, entropy, etc.<br>• Angular Second Moment (ASM):<br>$$ASM = \sum_{i=1}^{L}\sum_{j=1}^{L}[P(i,j)]^2$$<br>• Contrast:<br>$$Contrast = \sum_{n=0}^{L-1} n^2 \left\{ \sum_{\substack{i=1 \\ \|i-j\|=n}}^{L} \sum_{j=1}^{L} P(i,j) \right\}$$<br>• Correlation (Corr):<br>$$Corr = \frac{\sum_{i=1}^{L}\sum_{j=1}^{L} ijP(i,j) - \mu_i(i)\mu_j(j)}{\sigma_x(i)\sigma_y(j)}$$<br>• Sum of squares:

		$SumSquares = \sum_{i=1}^{L}\sum_{j=1}^{L}(i-\mu)^2 P(i,j)$<br>• Homogeneity:<br>$Homogeneity = \sum_{i=1}^{L}\sum_{j=1}^{L}\frac{P(i,j)}{1+\lvert i-j\rvert}$<br>• Inverse Difference Moment (IDM):<br>$IDM = \sum_{i=1}^{L}\sum_{j=1}^{L}\frac{P(i,j)}{1+\lvert i-j\rvert^2}$<br>• Sum average (SA):<br>$SA = \sum_{i=2}^{2L}\left[iP_{x+y}(i)\right]$<br>• Sum entropy (SE):<br>$SE = -\sum_{i=2}^{2L}P_{x+y}(i)\log_2\left[P_{x+y}(i)\right]$<br>• Sum variance (SV):<br>$SV = \sum_{i=2}^{2L}(i-SE)^2 P_{x+y}(i)$<br>• Entropy:<br>$Entropy = -\sum_{i=1}^{L}\sum_{j=1}^{L}P(i,j)\log_2[P(i,j)]$<br>• Different Variance (DV):<br>$DV =$ variance of P_{x-y}<br>• Different Entropy (DE):<br>$DE = \sum_{i=0}^{N_g-1}P_{x-y}(i)\log_2\left[P_{x-y}(i)\right]$<br>• Informational measure of correlation 1 (IMC1):<br>$IMC1 = \frac{HXY-HXY1}{\max\{HX,HY\}}$

		• Informational measure of correlation 2 (IMC2): $IMC2 = \sqrt{1 - e^{-2(HXY2 - HXY)}}$<br>• Maximum Correlation Coefficient (MCC): $MCC = (Second\ largest\ eigenvalue\ of\ Q)^{\frac{1}{2}}$<br>$Q = \sum_{k=1}^{L} \frac{p(i,k)p(j,k)}{P_x(i)P_y(k)}$<br>• Maximal Probability (MP): $MP = \max\{P(i,j)\}$<br>• Cluster Tendency (CT): $CT = \sum_{i=1}^{L}\sum_{j=1}^{L}\left[i + j - \mu_x(i) - \mu_y(j)\right]^2 P(i,j)$<br>where:<br>$\mathrm{P(i,j)}$: the probability distribution matrix of co-occurrence matrix $\mathrm{M(i,j;d,\theta)}$,<br>L: the number of discrete intensity levels in the image,<br>μ: the mean of $\mathrm{P(i,j)}$,<br>$\mathrm{p_x(i) = \sum_{j=1}^{L} P(i,j)}$ is the marginal row probabilities,<br>$\mathrm{p_y(i) = \sum_{i=1}^{L} P(i,j)}$ is the marginal column probabilities,<br>μ_x: the mean of p_x,<br>μ_y: the mean of p_y,<br>σ_x: the standard deviation of p_x,<br>σ_y: the standard deviation of p_y,<br>$\mathrm{p_{x+y}(k) = \sum_{i=1}^{L}\sum_{j=1}^{L} P(i,j),\ i + j = k,}$ $\mathrm{k = 2,3,\ldots,2L}$,<br>$\mathrm{p_{x-y}(k) = \sum_{i=1}^{L}\sum_{j=1}^{L} P(i,j),\ \lvert i - j\rvert = k,}$

		$k = 0,1,\ldots,L-1$,<br>$HX = -\sum_{i=1}^{L} p_x(i)\log_2[p_x(i)]$ is the entropy of $p_x(i)$,<br>$HY = -\sum_{i=1}^{L} p_y(i)\log_2[p_y(i)]$ is the entropy of $p_{y(i)}$,<br>$H = -\sum_{i=1}^{L}\sum_{j=1}^{L} P(i,j)\log_2[P(i,j)]$ is the entropy of $P(i,j)$,<br>$HXY1 = -\sum_{i=1}^{L}\sum_{j=1}^{L} P(i,j)\log(p_x(i)p_y(j))$,<br>$HXY2 = -\sum_{i=1}^{L}\sum_{j=1}^{L} p_x(i)p_y(j)\log(p_x(i)p_y(j))$.

Reference

1. Jan, J.K. and J.D. Andrea, *Surface shape and curvature scales.* Image and Vision Computing, 1992. **10**(8): p. 557-565.
2. Napel, S.A., et al., *Automated retrieval of CT images of liver lesions on the basis of image similarity: method and preliminary results.* Radiology, 2010. **256**(1): p. 243-52.
3. Mallat, S. (1989), "A theory for multiresolution signal decomposition: the wavelet representation," IEEE Pattern Anal. and Machine Intell., vol. 11, no. 7, pp. 674–693.
4. Pesquet, J.C.; H. Krim, H. Carfatan (1996), "Time-invariant orthonormal wavelet representations," IEEE Trans. Sign. Proc., vol. 44, 8, pp. 1964–1970.
5. Davis, L.S. and A. Mitiche, *Edge detection in textures.* Computer Graphics and Image Processing, 1980. **12**(1): p. 25-39.
6. Mandelbrot, B., *How long is the coast of Britain? Statistical self-similarity and fractional dimension.* Science, 1967. **156**(3775): p. 636-638.
7. Kido, S., et al., *Fractal analysis of internal and peripheral textures of small peripheral bronchogenic carcinomas in thin-section computed tomography: comparison of bronchioloalveolar cell carcinomas with nonbronchioloalveolar cell carcinomas.* J Comput Assist Tomogr, 2003. **27**(1): p. 56-61.
8. Amadasun, M., *Texural Features Corresponding to Texural Properties.* IEEE Transactions on Systems, Man and Cybernetics, 1989. **19**(5): p. 1264-1274.
9. Fogel, I. and D. Sagi, *Gabor filters as texture discriminator.* Biological Cybernetics, 1989. **61**(2): p. 103-113.

10. Laws, K.I. *Rapid Texture Identification*. in *Image Processing for Missile Guidance*. 1980. San Diego: SPIE.
11. Ganeshan, B., et al., *Tumour heterogeneity in oesophageal cancer assessed by CT texture analysis: preliminary evidence of an association with tumour metabolism, stage, and survival.* Clin Radiol, 2012. **67**(2): p. 157-64.
12. Galloway, M.M., *Texture analysis using gray level run lengths.* Computer Graphics and Image Processing, 1975. **4**(2): p. 172-179.
13. Castleman, K.R., *Digital Image Prncessing*, ed. E. Cliffs. 1996, NJ: Prentice-Hall.
14. Haralick, R.M., K. Shanmugam, and I. Dinstein, *Textural Features for Image Classification.* IEEE Transactions on Systems, Man and Cybernetics, 1973. **3**(6): p. 610-621.
